# Neural Dynamics of Processing Probability Weight and Monetary Magnitude in the Evaluation of a Risky Reward

**DOI:** 10.3389/fpsyg.2019.00554

**Published:** 2019-03-26

**Authors:** Guangrong Wang, Jianbiao Li, Pengcheng Wang, Chengkang Zhu, Jingjing Pan, Shuaiqi Li

**Affiliations:** ^1^ Neural Decision Science Laboratory, Weifang University, Weifang, China; ^2^ Reinhard Selten Laboratory, China Academy of Corporate Governance, Business School, Nankai University, Tianjin, China; ^3^ Department of Economic and Management, Nankai University Binhai College, Tianjin, China; ^4^ School of Economics, Shandong University, Jinan, China; ^5^ Business School, Tianjin University of Economic and Finance, Tianjin, China

**Keywords:** probability weight, money magnitude, risky choice, neural dynamics, ERP

## Abstract

Risky decision-making involves risky reward valuation, choice, and feedback processes. However, the temporal dynamics of risky reward processing are not well understood. Using event-related brain potential, we investigated the neural correlates of probability weight and money magnitude in the evaluation of a risky reward. In this study, each risky choice consisted of two risky options, which were presented serially to separate decision-making and option evaluation processes. The early P200 component reflected the process of probability weight, not money magnitude. The medial frontal negativity (MFN) reflected both probability weight and money magnitude processes. The late positive potential (LPP) only reflected the process of probability weight. These results demonstrate distinct temporal dynamics for probability weight and money magnitude processes when evaluating a risky outcome, providing a better understanding of the possible mechanism underlying risky reward processing.

## Introduction

Risky decision-making, which involves trade-offs between lotteries with differing magnitude and uncertainty, is ubiquitous in everyday life. Therefore, when making decisions among risky rewards, it is necessary to evaluate the subjective value of each risky reward. The subjective value of a risky reward depends on its probability and magnitude (Tversky and Kahneman, 1981; Brown and Braver, 2007). Prospect theory, an influential model of risky decision-making, suggests that the subjective value of a risky outcome depends on gains or losses relative to status quo and probability weighting function (Kahneman and Tversky, 1979; Camerer, 2000).

The neurocognitive mechanisms underlying risky decision-making involve several processes: valuation, choice, and feedback (Rangel et al., 2008; Liu et al., 2012). Previous neuroscience studies focused on the choice and feedback processes of risky decision-making, but the neural correlates for valuation of risky rewards are not well understood (see review by Chandrakumar et al., 2018). The focus of this study is on the temporal dynamics of the valuation process of risky rewards using an event-related brain potential (ERP) technique.

Neuroimaging studies have demonstrated that a number of brain regions including the ventromedial prefrontal cortex, amygdala, insula, anterior cingulate cortex (ACC), striatum, parietal, and temporal cortices are implicated in risk processing (Paulus and Frank, 2006; Berns et al., 2008; Hsu et al., 2009; Blankenstein et al., 2018). Several studies investigated the neural correlates of probability and magnitude of a risky reward. Berns and Bell (2012) measured independently the neural responses to magnitude and probability of a risky outcome by displaying serially magnitude and probability information. They found that the ventral and dorsal striatum were involved in the processes of magnitude and probability, respectively. These results demonstrate a second-order decision process, in which participants integrate judgments instead of information. In a study by Smith et al. (2009), high reward elicited more activation in several brain regions including the insula, amygdala, and posterior cingulate cortex when other parameters were held constant, as opposed to low reward. Low-probability reward induced more activation in the ACC than high-probability reward when other parameters were held constant.

Eye-tracking methodology has been used to investigate processes in risky decision-making (Rayner, 1998; Glöckner and Herbold, 2011). These studies suggested that risky decision-making relied mainly on automatic-intuitive processes, which were partially accounted for by automatic integration or simple heuristic models (Glöckner and Herbold, 2011; Fiedler and Glöckner, 2012; Venkatraman et al., 2014; Aimone and Ball, 2016). Eye-tracking studies have focused on risky choices in which two gambles were displayed simultaneously. Such paradigms did not allow us to distinguish valuation and choice processes. Furthermore, in real world, individuals usually face risky choice options serially.

Existing event-related brain potential (ERP) studies of risky decision-making focused on responses to risk-related decision and feedback. Only a minority of ERP studies have focused on the neural response to risky options (see review by Chandrakumar et al., 2018). Both feedback-related negativity (FRN) and P300 are two important ERP components involved in the risk process. The FRN, which is often known as reward positivity (RewP) associated with outcomes processing in the context of gains in contrast with losses (Foti et al., 2012; Proudfit, 2015; Yaple et al., 2018a,b), is larger following negative feedback relative to positive feedback (Wu and Zhou, 2009; Polezzi et al., 2010; Yang and Zhang, 2011; Yang et al., 2015; Zhao et al., 2016; Kardos et al., 2017). The P300, which is thought to reflect the outcome of stimulus evaluation and decision-making, was pronounced in response to the selection of a risky option and positive feedback (Yeung and Sanfey, 2004; Sato et al., 2005; Oberg et al., 2011; Schuermann et al., 2012; Wang et al., 2015).

While previous ERP studies have yielded important insights into the neural mechanisms of risky decision-making, there are limitations. First, risky decision-making involves valuation and choice processes, with evaluation of a risky reward most relevant to the valuation process (Rangel et al., 2008; Liu et al., 2012). Previous ERP paradigms, in which the participants’ task was to decide whether or not to accept a risky bet, did not allow one to distinguish among valuation and choice processes. Furthermore, evaluation of a risky reward involves its probability weight and magnitude, but previous ERP studies did not focus on these two components.

In this study, we developed a risky choice task to investigate neural mechanisms underlying probability weight and magnitude of risky rewards based on study paradigms derived from the intertemporal choice literature (Pine et al., 2009; Xia et al., 2017), given that there are a number of similarities between delay and probability discounting (Green and Myerson, 2004; Madden and Bickel, 2010; McKerchar and Renda, 2012). In our paradigm, each choice consisted of two risky options, which were first presented serially, then presented simultaneously. This allowed us to separate risky rewards valuation and selection processes. By controlling for the effects of probability and magnitude respectively, we could explore the neural mechanisms underlying probability weight and magnitude during evaluation of a risky reward.

According to previous research, several ERP components are associated with magnitude and probability processes. Based on these, we analyzed the ERP response related to probability weight and money magnitude in the evaluation of a risky reward. Since frontal P200 may be involved in stimulus evaluation and quick assessment (Boudreau et al., 2009; Lau et al., 2013), a hypothesis, in which the frontal P200 would reflect the difference between high- and low-probability rewards, was proposed. A second evaluated component was medial frontal negativity (MFN)^1^, which represents the early appraisal of feedback and is more pronounced for bad outcomes compared to good outcomes (Hajcak et al., 2006; Holroyd et al., 2006; Hewig et al., 2007; Boksem and de Cremer, 2010; Huang and Yu, 2014; Umemoto et al., 2017). In this study, when the magnitude of options was held constant, the high-probability rewards were considered “good” outcomes compared to low-probability rewards. Therefore, we predicted that the MFN would reflect the difference between high- and low-probability rewards. Similar predictions for the magnitude of risky rewards were made. Furthermore, the P300 has been shown to be sensitive to outcome evaluation, including the magnitude and valence of rewards (Goyer et al., 2008; Wu and Zhou, 2009; Harris et al., 2013; Righi et al., 2014). It is possible that the P300 would also encode the probability weight of risky rewards. Therefore, we hypothesized that the P300 or a later component would reflect the process of probability weight and money magnitude.

## Materials and Methods

### Participants

A total of 20 right-handed undergraduates were recruited. Twelve females and eight males participated. They were 20–25 years of age with a mean age of 22.35 (*SD* = 1.59). All participants had normal or corrected-to-normal visual acuity and no history of neurological or mental disease. All subjects signed an informed consent prior to the experiment, which was performed in accordance with the Declaration of Helsinki and was approved by the Ethics Committee of Reinhard Selten Laboratory, Nankai University. The participants received an average of 65 Chinese yuan (approximately $10) (Krajbich et al., 2012; Li et al., 2016; Yaple et al., 2017, 2018a,b).

### Task and Stimuli

According to Prospect Theory (Kahneman and Tversky, 1979), the subjective value (*V*) of a risky gamble is given by:

$$V=\overset{n}{\underset{i=1}{\sum }}\pi \left(p_{i}\right)u\left(x_{i}\right)$$

In the present study, we focused on the evaluation of risky rewards. Therefore, each risky option consisted of a risky reward and a zero reward. Therefore, the subjective value (*V*) of a risk option was expressed as:

V=π(p)×u(x)

The function *π(p)* represents the subjective probability to objective probability *p*, with *u(x)* the undiscounted utility of a reward (*x*).

This study tried to explore neural processing of probability weight and money magnitude of a risky reward. Since the subjective value of a risky reward is determined by the magnitude and probability of its receipt, an experimental paradigm was designed to allow comparison based on: (1) different probabilities but same money magnitude, and (2) different money magnitude but same probability. To obtain subjective utility related to probability, two types of stimuli were considered: winning CNY50 at the probability of 0.2 (low probability, LP) and CNY50 at the probability of 0.5 (high probability, HP). Similarly, for money magnitude, we considered two types of stimuli: winning CNY15 at the probability of 0.66 (small magnitude, SM) and CNY40 at the probability of 0.66 (large magnitude, LM). In addition, in order to improve the reality of the experiment and decrease the risk that participants will be bored, some stimuli including CNY10 by 0.99, CNY30 by 0.33, CNY60 by 0.33, and CNY20 by 0.99 were defined as filling materials.

The task of the participants was to choose between two options with different magnitude and probability of occurrence. Each option of a choice was presented serially to separate decision-making and option valuation processes. At the end of the experiment, two of the participant’s choices were selected at random and used for subject payment.

### Procedure

The rules of the experimental task were instructed to the participants by explaining written instructions. The task was performed in a quiet and isolated laboratory. The participants were told that they would be paid for participation after completion of the experiment. The recording session took approximately 30 min.

**Figure 1:**
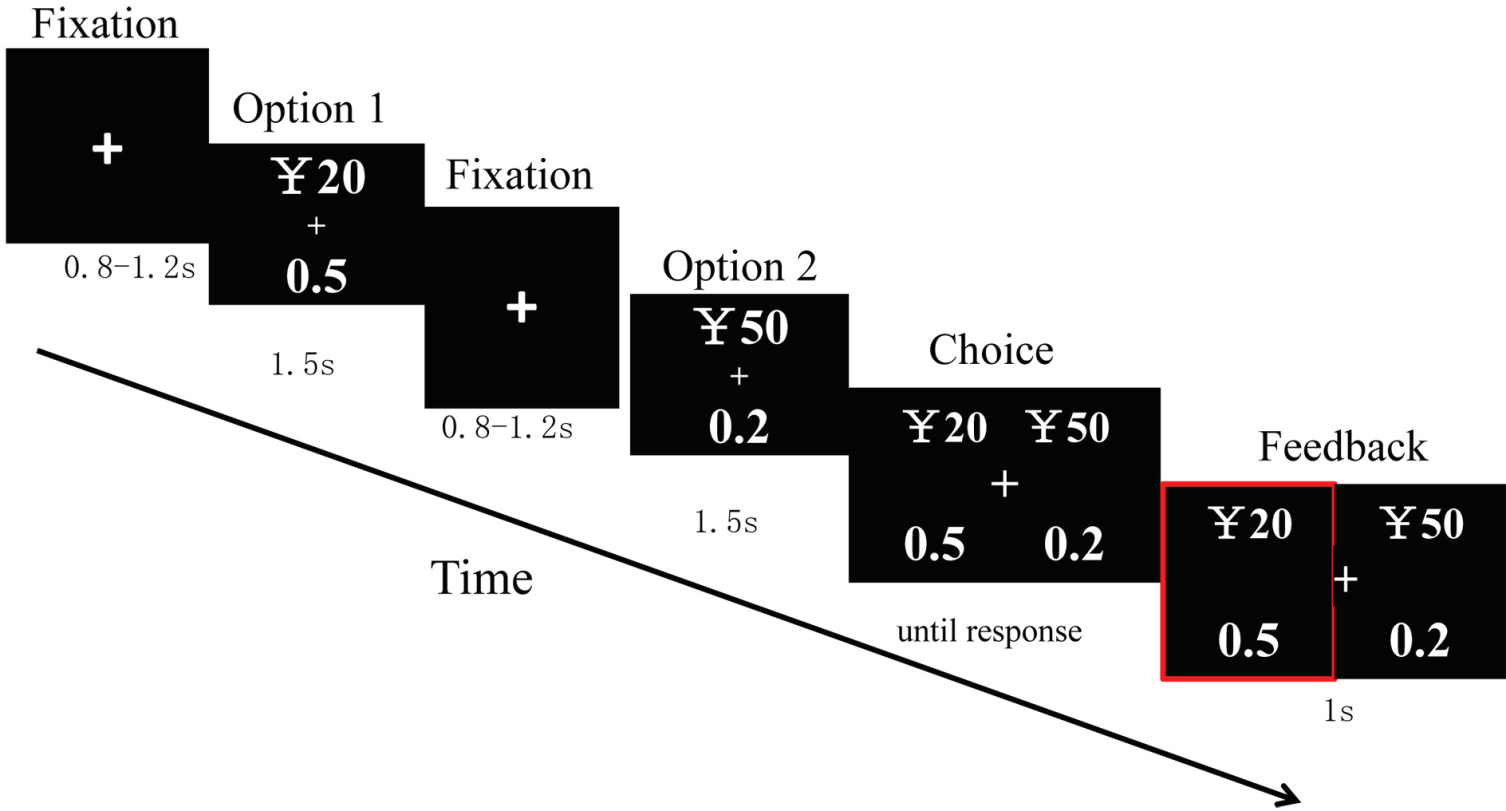
Sequence of trail events.

After 8 practice trials, a total of 100 trials were randomly divided into 2 blocks with 50 trials each. Each trial was created through the following sequence. In each trial, a cross was first displayed in the center of a screen for 800–1,200 ms. Afterward, option 1 was presented for 1,500 ms. Then, after a cross of 800–1,200 ms, option 2 was presented for 1,500 ms. Next, the choice was displayed until a response had been made. The presentation of the two options for each type of stimuli was counterbalanced in a random order across trials. Then, their choice was shown for 1,000 ms, after which a blank screen was displayed for 1,000 ms, and then the next trial started (Figure 1).

### Electroencephalography (EEG) Recording and Analysis

EEGs were continuously acquired at a 1,000 Hz sampling rate with a Neuroscan Synamp2 Amplifier, by using an electrode cap with Ag/AgCl electrodes mounted according to the extended international 10–20 system. The EEG signals were amplified online (band pass: 0.05–100 Hz). All rows of electrode recordings were referenced online to the left mastoid, and they were re-referenced offline to the average of the left and right mastoid. Electrode impedance was kept under 5 kΩ. Following the electrode application, the participants sat in a comfortable chair located in a shielded room and were asked to fix on the center of the computer display located 1 m away from their eyes during the experiment.

EEG epochs of 1,000 ms (from −200 to 800 ms after the onset of stimulus) were extracted offline, and the 200-ms pre-stimulus defined as baseline. Ocular artifacts were corrected. Trials contaminated by amplifier clipping, bursts of electromyographic activity or peak-to-peak deflection exceeding ±75 μV were excluded from further analysis. The remaining trials were baseline corrected. The EEG segments were averaged separately for probability type (HP vs. LP) and magnitude type (LM vs. SM). Averaged ERPs were digitally filtered with a low-pass filter at 30 Hz. Within-subject repeated measure analysis of variance (ANOVA) were used to analyze ERP data. Behavior and ERP data were statistically analyzed using SPSS (version 22, SPSS Inc., Chicago, IL, USA). A Greenhouse-Geisser correction for violation of sphericity assumption was applied when the degrees of freedom were more than one. The significance level was set at 0.05 for all analyses. To control for family-wise error for multiple *t*-tests, *p* were Bonferroni corrected.

Based on the visual inspection of the grand-average waveforms, three components were analyzed. The frontal P200 was measured as peak amplitude between 150 and 250 ms after stimulus onset at F3, Fz, F4, FC3, FCz, and FC4 (Polezzi et al., 2008; Molinaro and Carreiras, 2010; Gui et al., 2016). The MFN component was measured as peak amplitude between 250 and 350 ms after stimulus onset at F3, Fz, F4, FC3, FCz, and FC4 (Boksem and de Cremer, 2010; Schuermann et al., 2012; Huang and Yu, 2014; Xia et al., 2017). The LPP was measured as mean amplitude between 450 and 650 ms after stimulus onset at CP3, CPz, CP4, P3, Pz, and P4 (Harris et al., 2013; Righi et al., 2014; Gui et al., 2016). ERP analyses were conducted by repeated-measure ANOVAs, with electrode (for P200 and MFN: F3, Fz, F4, FC3, FCz, and FC4, for LPP: CP3, CPz, CP4, P3, Pz, and P4) and probability (high, low), and electrode and magnitude (large, small), respectively.

## Results

### Behavioral Results

Behavioral results are shown in Table 1. For the choice of CNY50 by 0.2 probabilities and CNY15 by 0.66 probabilities, 44.60% of decisions chose the former. For the choice of CNY50 by 0.5 probabilities and CNY40 by 0.66 probabilities, 38.73% of decisions chose the former. The average response time was 775.98 ms (*SD* = 611.5195) and 719.52 ms (*SD* = 413.1792), respectively. For the choice of CNY50 by 0.2 probabilities and CNY40 by 0.66 probabilities, all decisions chose the latter. For the choice of CNY50 by 0.5 probabilities and CNY15 by 0.66 probabilities, 95.24% of decisions chose the former. The average response time was 550.69 ms (*SD* = 204.8855) and 590.89 ms (*SD* = 268.96), respectively.

**Table 1:** Behavioral results.

Choice type (option 1: option 2)	Percentage of option 1	Response time	Standard deviation
CNY 50 by 0.2: CNY 15 by 0.66	44.60%	775.98	611.5195
CNY 50 by 0.2: CNY 40 by 0.66	0.00%	550.69	204.8855
CNY 50 by 0.5: CNY 15 by 0.66	95.24%	590.89	268.9600
CNY 50 by 0.5: CNY 40 by 0.66	38.73%	719.52	413.1762

Participants took more response time to make decision between the choices in which the expected value of two options was similar, compared to choices in which there was large difference between the expected values of two options (*p* = 0.02). Based on formal logic, when the expected value of each option of a risky choice is similar, the higher level of conflict requires more brain resources for conflict resolution, which results in more response time.

Behavioral data showed that participants chose the option with largest expected value. This is consistent with previous studies and demonstrates that participants clearly understood the experimental task.

### ERP Results for Probability Weight

#### P200

**Figure 2:**
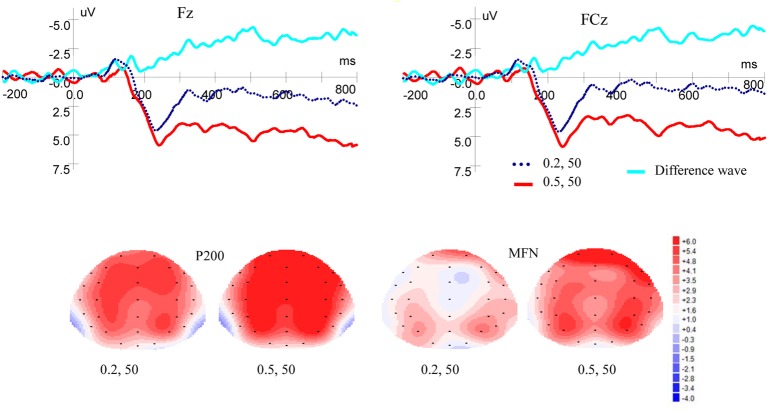
Grand-averaged ERP waves at electrodes Fz and FCz for probability and topographic maps for P200 and MFN.

Figure 2 shows ERP waveforms and topographic maps for probability processes at Fz and FCz electrodes. In the frontal area, there was a significant main effect of P200 for probability levels [*F*(1, 19) = 8.309, *p* = 0.010, *η*^2^ = 0.304], no main effect for laterality [*F*(2, 38) = 3.899, *p* = 0.051, *η*^2^ = 0.170], and no interaction between probability levels and laterality [*F*(2, 38) = 2.160, *p* = 0.145, *η*^2^ = 0.102] were found. In the frontal-central scalp area, significant main effects were found for probability levels [*F*(1, 19) = 7.586, *p* = 0.013, *η*^2^ = 0.285] and laterality [*F*(2, 38) = 5.117, *p* = 0.017, *η*^2^ = 0.212]. There was no interaction between probability levels and laterality [*F*(2, 38) = 2.288, *p* = 0.126, *η*^2^ = 0.107]. High-probability rewards elicited more positive P200 than low-probability ones when the magnitude was kept constant.

#### MFN

As shown in Figure 2, in the frontal area, significant main effects of MFN were observed for probability levels [*F*(1, 19) = 10.389, *p* = 0.004, *η*^2^ = 0.353] and laterality [*F*(2, 38) = 5.490, *p* = 0.024, *η*^2^ = 0.224], but no interaction was found between probability levels and laterality [*F*(2, 38) = 1.656, *p* = 0.211, *η*^2^ = 0.080]. In the frontal-central area, significant main effects of MFN were observed for probability levels [*F*(1, 19) = 12.067, *p* = 0.003, *η*^2^ = 0.388] and laterality [*F*(2, 38) = 5.443, *p* = 0.020, *η*^2^ = 0.223]. There was no interaction between probability levels and laterality [*F*(2, 38) = 3.594, *p* = 0.053, *η*^2^ = 0.159]. These results showed that low-probability rewards elicited more negative MFN than high-probability ones, when the magnitude was kept constant.

#### LPP

**Figure 3:**
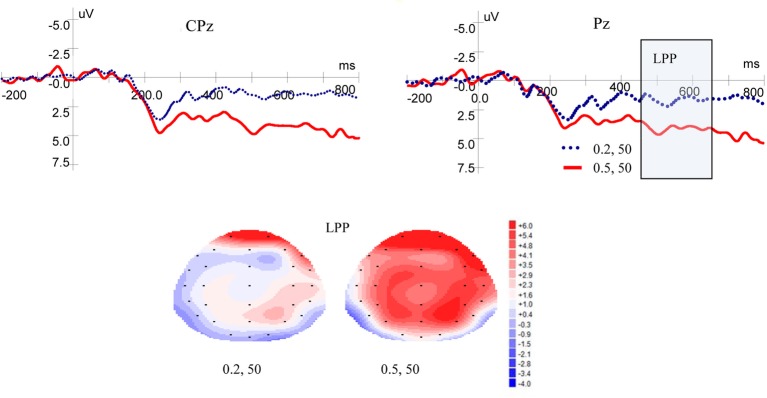
Grand-averaged EPR waveforms at electrodes Pz and CPz for probability, and topographic maps for LPP.

Figure 3 shows ERP waveforms and topographic maps for probability processes at Pz and CPz electrodes. In the parietal area, a significant main effect for LPP was found for probability levels [*F*(1, 19) = 17.599, *p* = 0.000, *η*^2^ = 0.481]. There were no significant main effect for laterality [*F*(2, 38) = 2.418, *p* = 0.105, *η*^2^ = 0.113] and no interaction between probability levels and laterality [*F*(2, 38) = 0.032, *p* = 0.935, *η*^2^ = 0.002]. In the central-parietal area, significant main effects for LPP was found for probability levels [*F*(1, 19) = 19.374, *p* = 0.000, *η*^2^ = 0.505] and laterality [*F*(2, 38) = 3.884, *p* = 0.043, *η*^2^ = 0.170], but no interaction was found between probability levels and laterality [*F*(2, 38) = 0.033, *p* = 0.934, *η*^2^ = 0.002]. These results demonstrated that high-probability rewards elicited more positive LPP than low-probability ones, when the magnitude was kept constant.

### ERP Results for Money Magnitude

#### P200

**Figure 4:**
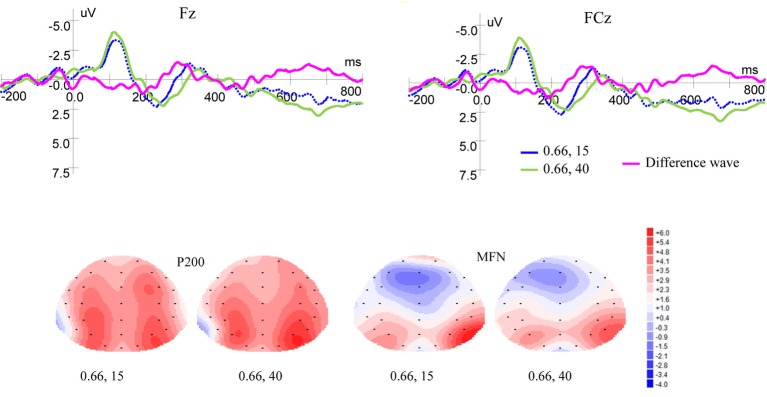
Grand-averaged EPR waveforms at electrodes Fz and FCz for magnitude, and topographic maps for P200 and MFN.

Figure 4 shows ERP waveforms and topographic maps for money magnitude at Fz and FCz electrodes. In the frontal area, there were no significant P200 effect for reward magnitude [*F*(1, 19) = 0.093, *p* = 0.764, *η*^2^ = 0.005] and no interaction between magnitude and laterality [*F*(2, 38) = 0.045, *p* = 0.921, *η*^2^ = 0.002]. But there was significant P200 effect for laterality [*F*(2, 38) = 7.140, *p* = 0.002, *η*^2^ = 0.273]. In the frontal-central area, no significant P200 effects were observed for magnitude levels [*F*(1, 19) = 0.095, *p* = 0.761, *η*^2^ = 0.005] and laterality [*F*(2, 38) = 1.716, *p* = 0.198, *η*^2^ = 0.083]. There was no interaction between magnitude levels and laterality [*F*(2, 38) = 0.059, *p* = 0.878, *η*^2^ = 0.003].

#### MFN

As shown in Figure 4, in the frontal area, significant main effects for MFN were found for magnitude levels [*F*(1, 19) = 6.380, *p* = 0.021, *η*^2^ = 0.251] and laterality [*F*(2, 38) = 13.866, *p* = 0.000, *η*^2^ = 0.422], but no interaction was found between magnitude levels and laterality [*F*(2, 38) = 1.461, *p* = 0.246, *η*^2^ = 0.071]. In the frontal-central area, significant main effects for MFN were found for magnitude levels [*F*(1, 19) = 5.619, *p* = 0.029, *η*^2^ = 0.228] and laterality [*F*(2, 38) = 9.404, *p* = 0.001, *η*^2^ = 0.331], but no interaction was found between magnitude levels and laterality [*F*(2, 38) = 0.735, *p* = 0.454, *η*^2^ = 0.037]. Given same probability weight, small rewards elicited more positive MFN than large ones.

#### LPP

**Figure 5:**
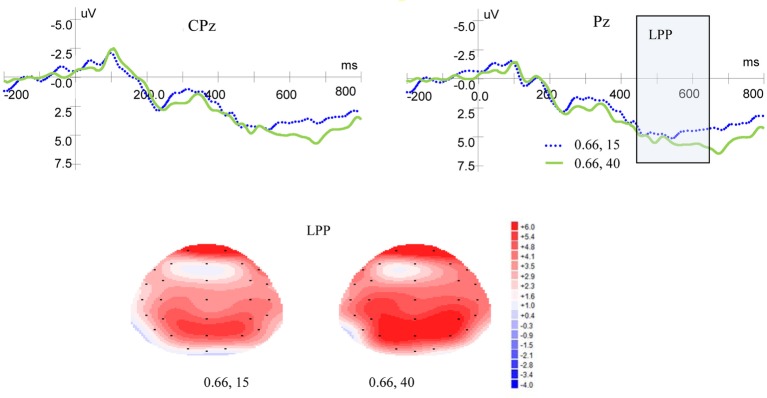
Grand-averaged EPR waves at electrodes Pz and CPz for magnitude, and topographic maps for LPP.

Figure 5 shows ERP waveforms and topographic maps for magnitude at Pz and CPz electrodes. In the parietal area, no significant main effect of LPP was found for magnitude levels [*F*(1, 19) = 1.937, *p* = 0.180, *η*^2^ = 0.093] and no interaction was found between magnitude levels and laterality [*F*(2, 38) = 1.867, *p* = 0.176, *η*^2^ = 0.089]. There was no significant main effect for laterality [*F*(2, 38) = 1.651, *p* = 0.208, *η*^2^ = 0.080]. In the central-parietal area, no significant main effects of LPP were found for magnitude levels [*F*(1, 19) = 1.431, *p* = 0.246, *η*^2^ = 0.070] or laterality [*F*(2, 38) =1.804, *p* = 0.186, *η*^2^ = 0.087], and no interaction was found between magnitude levels and laterality [*F*(2, 38) = 0.676, *p* = 0.473, *η*^2^ = 0.034].

### Relationships Among Evaluation and Risky Decision-Making Behaviors

In the present study, there were two types of normal choices: CNY50 by 0.2 probabilities (expected value (*EV*) = 10) and CNY15 by 0.66 probabilities (*EV* = 9.9), CNY50 by 0.5 probabilities (*EV* = 25) and CNY40 by 0.66 probabilities (*EV* = 26.4). Based on behavioral results, participants were divided into groups. For the choice of CNY50 by 0.2 and CNY15 by 0.66, 9 participants (LH) almost chose the former and 11 participants (LL) almost chose the latter. For the choice of CNY50 by 0.5 probabilities and CNY40 by 0.66 probabilities, 8 participants (HH) almost chose the former and 12 participants (HL) almost chose the latter.

**Figure 6:**
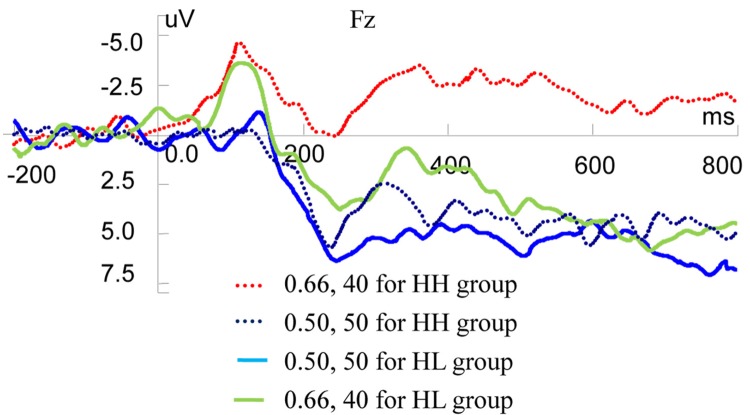
Grand-averaged EPR waves at electrode Fz for different groups (HH, HL).

We conducted independent *t*-test using ERP data on Fz to analyze the correlation among valuation of risky rewards and risky decision-making behaviors (Figure 6). Statistical results showed that there was no significant ERP difference between LH and LL groups when they observed CNY50 by 0.2 and CNY15 by 0.66, respectively. However, when observing CNY50 by 0.5 and CNY40 by 0.66 respectively, HH and HL groups displayed different ERP valuation. For CNY50 by 0.5, there was no significant ERP difference between HH and HL groups, but for CNY40 by 0.66, two types of participants expressed different valuation. For P200 component, the mean amplitudes were 1.1040 and 4.9620 μV in HH and HL groups, respectively (*t* = −2.025, *df* = 18, *p* = 0.058). For MFN component, the mean amplitudes were −4.0098 and 0.2311 μV in HH and HL groups, respectively (*t* = −1.675, *df* = 18, *p* = 0.111). For the LPP component, the mean amplitudes were −1.8682 and 4.5597 μV in HH and HL groups, respectively (*t* = −2.437, *df* = 18, *p* = 0.025).

## Discussion

Risky decision-making involves risky reward valuation, choice, and feedback processes. This study focused on risky reward valuation. This investigation assessed the neural dynamics involved in the processing of probability weight and money magnitude. ERP results demonstrated distinct temporal dynamics for probability weight and money magnitude processes. The early frontal P200, MFN, and LPP components all represented the process of probability weight; however, only the MFN component was associated with the process of money magnitude when evaluating a risky reward.

Frontal P200 revealed a significant main effect of probability weight on the frontal and frontal-central areas, but no significant main effect of money magnitude for a risky reward. Low-probability reward elicited less positive P200 amplitude when compared to high-probability reward at the same magnitude. Previous studies showed that P200, the probable sources of which may be the mesotelencephalic dopamine reward system, likely associates with stimulus evaluation and quick assessment (Boudreau et al., 2009; Chen et al., 2009). The P200 component has been shown to be involved in attention to relevant cues including reward-related stimuli (Molinaro and Carreiras, 2010; Lau et al., 2013; Gui et al., 2016). Several ERP studies, which explored the processing of reward, found that a reward condition elicited larger P200 compared to a non-reward condition (Martin and Potts, 2004; Franken et al., 2010). The relationships between low- and high-probability rewards were similar to those relationships. Schuermann et al. (2012) found that P200 was enhanced on negative feedbacks in high-risk compared to low-risk choices, which suggests that large negative prediction errors are already processed in the P200 time range. Hence, our findings are consistent and suggest that participants detected the initial feature of probability, not magnitude at the early stage of risky option processing.

The MFN component, which reflects the impact of dopamine-dependent reward signals on the ACC, may represent the evaluation of reward value (Gehring, 2002; Holroyd and Coles, 2002; Proudfit, 2015). In the present study, consistent with this classical theory, the MFN component showed significant main effects of both probability weight and money magnitude of a risky reward. Given the same magnitude, low-probability options evoked a more negative MFN as compared to high-probability options. Moreover, small magnitude induced a more pronounced MFN than large magnitude for the same probability weight. Existing studies demonstrated that the MFN component reflects the early appraisal of feedback, in which the MFN response to unfavorable outcomes is larger compared to favorable outcomes (Holroyd et al., 2006; Goyer et al., 2008; Boksem and de Cremer, 2010; Broyd et al., 2012; Huang and Yu, 2014; Umemoto et al., 2017). Our results are consistent with those findings. Since risky decision-making is ubiquitous, high-probability rewards are considered better than low-probability ones for the same magnitude. In other words, a high-probability reward is a “good” outcome, relative to a low-probability reward when the magnitude is constant. Likewise, large rewards are considered better than small rewards with the same probability weight.

The LPP component has been mainly associated with affective and emotional processing (Ferrari et al., 2011; Righi et al., 2012). Many studies have found positive and negative stimuli to elicit larger LPP amplitude than neural stimuli, which suggests that more brain resources are allocated to affective stimuli (Foti and Hajcak, 2008; Hua et al., 2014; Zhang et al., 2014; Guo et al., 2018). In this study, LPP was more positive for high-probability than low-probability reward, demonstrating that participants paid more attention to high-probability reward. The study of Harris et al. (2013) found that LPP reflected process differences between liked and disliked food items. Those results suggest LPP is related to valuation modulation. The relationship between liked and disliked foods is similar to that between high- and low-probability rewards. Wu et al. (2011) investigated the neural response to selection of risky rewards. They found that medial prefrontal cortex (mPFC) involved in the process of magnitude, and mPFC and ACC correlated with probability. Given that P300 and LPP amplitude variation is related to the striatum (Pfabigan et al., 2014) and the MFN is correlated with ACC and mPFC (Gehring, 2002; Boksem and de Cremer, 2010), their findings support our conclusions.

In summary, this study investigated neural dynamics of the processes associated with probability weight and money magnitude in the evaluation of a risky reward. ERP results demonstrated P200, MFN, and LPP components to reflect the processing of probability weight, while only the MFN component reflected the processing of money magnitude when evaluating a risky reward. These findings contribute to an understanding of the temporal course of processing probability weight and money magnitude during risky choices.

## Author Contributions

GW and JL conceived and designed this study. GW, JL, PW, and CZ designed experimental stimuli and procedures. CZ and JP implemented experimental protocols and collected data. SL and GW analyzed data. GW and JL wrote the paper.

### Conflict of Interest Statement

The authors declare that the research was conducted in the absence of any commercial or financial relationships that could be construed as a potential conflict of interest.

## Footnotes

^1^The MFN is a class of ERP components which include the FRN and ERN. The MFN has been proposed to reflect a motivational/affective evaluation of negative outcomes. Both feedback and stimulus information elicit MFN. The MFN in the context of feedback-locked ERPs could reflect whether outcomes matched expectations, while the MFN in the context of stimulus-locked ERPs could reflect whether the events violated (social and non-social) expectancy (Holroyd and Coles, 2002; Goyer et al., 2008; Boksem and de Cremer, 2010; Schuermann et al., 2012). This study focused on stimulus-locked ERPs.
